# Colorectal cancer screening behaviors

**DOI:** 10.7717/peerj.10951

**Published:** 2021-03-08

**Authors:** Selda Tekiner, Gülsen Ceyhun Peker, Mine Ceylan Doğan

**Affiliations:** 1 Family Medicine Department/Faculty of Medicine, Ankara University, Ankara, Türkiye; 2 Ufuktepe Family Health Center, Ministry of Health, Ankara, Türkiye

**Keywords:** Primary care, Colorectal cancer, Health belief model, Screening

## Abstract

**Introduction:**

Colorectal cancer screening program compliance in Turkey is around 20–30%. Factors that may affect preventive health behavior, mainly individuals’ values, beliefs and attitudes, should be explored. A “Health Belief Model” (HBM) scale was developed in 1950 to explain the insufficient participation of some individuals in screening programs. The scale was adopted for colon cancer in 2002. The validity and reliability study of this scale for the Turkish language was conducted in 2007. In this study we aim to evaluate the health beliefs of relatively young individuals before the age of screening in relation to attitudes to colorectal cancer screening via the Turkish version of the scale.

**Materials and Methods:**

A questionnaire composed of 14 sociodemographic questions and 33 HBM scale questions were applied to the patients of a Family Medicine outpatient clinic where the majority of the patients are relatively young people. The data was analyzed using the IBM SPSS version 21.0.

**Results:**

A total of 310 subjects at the age of 18 and over were informed about the study. The study was completed with 215 subjects with a participation rate of 69.3%. The average age of the participants was 35.4 ± 12.8 years; 62.8% of them were women; 26.0% were students. 94.4% of the participants did not have a history of colon cancer among their first degree relatives. 58.1% agreed that “A colonoscopy should be done every ten years starting at age 50 to screen for colon cancer.” Age, marital status, education and occupation status were found to have an effect on barrier scores. Young participants, singles, and those with a primary and/or secondary school education had statistically significant higher barrier scores. The motivation scores of high school graduates were higher than university graduates. The seriousness scores of men were found to be higher than women.

**Conclusion:**

Our study points out that attitudes about preventive health measures are mainly associated with age, gender, education level and marital status. These personal characteristics should be taken into consideration while offering screening programs and preventive health measures to individuals in order to protect against colorectal cancer. It is better to give specific messages according to personal characteristics and specific barriers instead of general messages about conducting screening tests beginning from a young age.

## Introduction

According to the most recent reports of the WHO, Colorectal Cancer (CRC) is the 2nd most common cause of mortality from cancer globally with 862,000 deaths. In Turkey, CRC is the 3rd most common cancer type after lung and prostate cancer in men, and breast and thyroid cancer in women (Armelao et al., 2011). Early diagnosis is a crucial public health strategy in all settings especially in high risk populations, and effective screening methods are available for CRC. CRC screening is one of the few methods for decreasing CRC incidence and invasive cancer morbidity and mortality with a proven efficiency (Bénard et al., 2018; Levin et al., 2008; Soytürk, 2013).

Based on the currently available infrastructure and facilities in Turkey, every individual from the age of 50 is invited to have a colonoscopy every 10 years. A realizable target is public screening between 50 and 70 years of age. So, each individual older than 50 is invited to participate in a colorectal cancer screening via his/her own family physician. For the CRC screening programs to reach the desired outcomes the participation rate should be at least 70%, but in Turkey it is around 20–30% (Armelao et al., 2011; Sağlık Bakanlığı, 2013). Increasing public awareness in order to bring about public consciousness and behavioral changes carries a very important role in the realization of this purpose (Goldbohm et al., 1994; Alberts et al., 2000; Gimeno Garcia et al., 2014). In an effort to explain the insufficient participation of some individuals in prevention and screening programs while other people adopt preventive health behaviors, Hochbaum, Kegeles, Leventhal and Rosenstock developed the “Health Belief Model” (HBM) scale in 1950 (Hochbaum, 1958; Rosenstock, 1960). They identified that age, gender, socioeconomic status and ethnicity have an effect on preventive health behaviors. However, even if health services are provided for free, people with a lower socioeconomic status use this service less often. This observation suggests that other factors also affect preventive health behaviors, namely the individuals’ values, beliefs and attitudes. If we can identify problematic beliefs and attitudes, we can personalize health education and treatments for the individual accordingly (Gözüm & Çapık, 2014). Attitudinal components of health related behaviors are important. If attitudes related to health behaviors can be identified, interventions for attitude changes can be developed and an increase in desirable health behaviors would result (Yılmaz, Dereli & Yelten, 2016).

The Health Belief Model scale was adapted by Champion for breast cancer in 1984 and it was emphasized that it can also be used for other cancers by revising some items (Champion, 1984; Champion & Scott, 1997). Then in 2002 Jacobs adapted the scale to colorectal cancer by changing a few questions and changing the word “breast cancer” in each of its six dimensions to “colon cancer” (Jacobs, 2002; Aydoğdu & Bahar, 2011). The validity and reliability study of this scale for the Turkish language was conducted by Ozsoy, Ardahan & Ozmen (2007). They showed that the Turkish version of the Champion’s Health Belief Model Scales has good structural characteristics and is a reliable and valid instrument that can be used to measure beliefs related to colorectal cancer. However, they stated that there is a need to test these scales with different groups. In this study we aimed to assess the health beliefs of people before the age of screening in relation to attitudes to colorectal cancer screening via the Turkish version of this scale.

Our hypothesis for this research was that this group’s health beliefs about colon cancer screening were not as desired, and that women have more developed health beliefs in regard to preventing colon cancer.

## Materials and Methods

This study was carried out in the Ankara University Family Medicine policlinic. The pool of people going to the policlinic are mostly the young and middle age group, and of female gender. Hence, most of our patients are not at the recommended age for colorectal cancer screening. However, an early intervention is essential in order to educate the society and change health misbeliefs.

Based on the health belief scale scores of the participants in the article, “Some sociodemographic characteristics, healthy life style behaviors and health beliefs of individuals aged 50 and over effect on screening behaviors of colon cancer,” it was calculated that at least 199 people should be included in the study at a 0.20 effect size (*d* = 0.20) with 80% power and α = 0.05 error level (Yılmaz, Dereli & Yelten, 2016). The G Power 3.1.9.2 Package program was used for calculation.

We prepared a form containing sociodemographic data and asking for some CRC information. We asked the participants’ weight and height, nutritional habits and some thoughts and knowledge about CRC. This 14-item form was presented to the participants first. Then the “Health Belief Model” scale consisting of 33 items was administered. A written informed consent form was obtained from the participants.

The study was approved by the Ethics Committee of Ankara University (Ankara, Turkey) for non-clinical research with a reference number of 14/223.

### Data collection

All data was collected by the same research assistant from July 2016 to October 2016.

The researcher distributed an informed consent form to everyone above age 18 who came to the Family Physician policlinic, and she carried the study out on those who volunteered. She first introduced the questionnaire with 14 questions including CRC information, as well as sociodemographic characteristics. Then the researcher carried out the Turkish language version of the “Health Belief Model Scale” for colon cancer.

The health belief model is a usable model for evaluating healthy individuals’ health behaviors and their beliefs and attitudes regarding CRC. It is also useful in organizing information about clients’ views on the state of their health and what factors may influence them to change their behavior. The Health Belief Model, when used appropriately, provides organized assessment data about clients’ abilities and motivation to change their health status.

### The health belief model scale

The health belief model is one of the oldest models developed by adapting behavioral science theory to the field of health. It is a model frequently used in health behavior practices today. The health belief model explains the relationship between a person’s beliefs and behaviors, and the effect of individual motivation on health behaviors at the individual decision-making level. Champion developed the health belief model scale for breast cancer, and Jacobs adapted this scale to colorectal cancer, replacing several of the scale’s questions, as well as the word “breast cancer” in each of its six dimensions to colon cancer. The validity and reliability study of the scale for the Turkish language was conducted by Ozsoy, Ardahan & Ozmen (2007). The Turkish version of the HBM scale used in this study with permission from Özsoy, consists of a total of 33 items clustered into five subscales: confidence, benefits, and health motivation (first subscale = 11 items); susceptibility (second subscale = 7 items); barriers (third subscale = 6 items); health motivation (fourth subscale = 5 items); and seriousness (fifth subscale = 6 items). The subscales are evaluated independently from each other.

Items were formatted with a 5-point Likert scale, from “strongly agree” to “strongly disagree”. “I strongly disagree” is 1 point, “I disagree” is 2 points, “I somewhat agree” is 3 points, “I agree” is 4 points and “I strongly agree” is designated 5 points. The minimum (min) and maximum (max) points are 11–55 for benefits, 6–30 for susceptibility, 6–30 for barriers, 5–25 for motivation and 5–25 for seriousness.

Increased scores mean increased confidence and benefits, increased susceptibility, increased motivation and increased seriousness. Increased score barriers mean the person perceives the barriers to be higher.

The Cronbach alpha values of the scale were between 0.54 and 0.88. Confidence and benefits: 0.88; Susceptibility: 0.76; Barriers: 0.60; Health Motivation: 0.54; Seriousness: 0.58 (Ozsoy, Ardahan & Ozmen, 2007).

### Analysis

“IBM SPSS Version 21.0” was used in data analysis, *p* < 0.05 was accepted as the statistical significance limit.

Mean standard deviation, median, minimum and maximum values are given in descriptive statistics regarding continuous data. Percentage values are given in discrete data.

The Shapiro Wilk test was used to examine the appropriateness of scale scores in relation to a normal distribution. When comparing the scores of the Health Belief Model scale with independent variables, a *T* test and a Mann Whitney *U* Test were used for two-group independent variables; and a ANOVA and a Kruskal Wallis Variance analysis were used for independent variables with more than two groups. The group or groups the difference originated from were evaluated via the Tukey and Kruskal Wallis multiple comparison test.

## Results

During the study period, 310 subjects aged 18 and over were informed about the study of which 80 subjects did not agree to participate, 5 subjects aged 50 and over were excluded because they had knowledge about CRC and/or had colonoscopy and 10 did not complete the questionnaire. This current study included 215 subjects with a compliance rate of 69.3%.

The average age of participants was 35.4 ± 12.8 and 62.8% of them were women, 54.4% were married and about half of them were university graduates (50.2%). The Average Body Mass Index was 24.18 ± 5.54. 94.4% of the participants did not have a history of colon cancer among their first degree relatives. Sociodemographic characteristics of participants are given in Table 1.

**Table 1:** Sociodemographic characteristics of the participants.

Characteristic		*n*	%
Age	18–29	88	40.9
	30–39	49	22.8
	40–49	45	20.9
	50+	33	15.3
Sex	Woman	135	62.8
	Man	80	37.2
Marital status	Married	117	54.4
	Single	98	45.6
Education level	Secondary school/lower	24	11.2
	High school	83	38.6
	University	108	50.2
Employment	Working	121	56.3
	Student	56	26.0
	Retired	19	8.8
	Housewife + Unemployed	19	8.8
Health coverage	Yes	206	95.8
	No	9	4.2
Colon cancer history in first degree relative	Yes	12	5.6
	No	203	94.4
	Total	215	

A total of 79.12% of the participants stated that they did not use alcohol, 60% did not exercise, 51.6% stated that they were eating a diet poor in protein, fat and carbohydrates.

While 58.1% agreed that “A colonoscopy should be done every 10 years starting at age 50, in order to screen for colon cancer”, 31.6% had no idea and 10.3% disagreed.

The mean points of each statement of the HBM scale for protection against colon cancer are given in Table 2.

**Table 2:** Mean points of each statement of HBM scale for protection from colon cancer.

		Median	Min–Max
Confidence-benefit	1: I would like to determine my health problems early	1	1–5
	2: It is very important for me to stay healthy	1	1–5
	3: If necessary, I trust myself to have regular controls for early diagnosis of colon cancer	2	1–5
	4: Regular controls for early detection of colon cancer provides an opportunity to catch the cancer at an early stage	1	1–4
	5: I search for new information for being healthy	2	1–5
	6: I can continue regular controls if I have colon cancer	1	1–5
	7: I know the importance of things to do to stay healthy	1	1–5
	8: I can notice the normal and abnormal changes in my bowel habits	2	1–5
	9: My risk of dying from colon cancer will decrease if I have regular controls for early diagnosis of colon cancer	1	1–4
	10: My risk of having a big and unshapely operation (colostomy) if I have colon cancer will decrease if I have regular controls for early diagnosis of colon cancer	2	1–4
	11: I can catch colon cancer early if I have regular controls	1	1–4
Susceptibility	12: I will most likely have colon cancer in the future	4	1–5
	13: I can feel that I will have colon cancer in the future	4	1-5
	14: I have risk of having colon cancer in the next 10 years	4	1-5
	15: I have a high risk to have colon cancer	4	1–5
	16: My risk of having colon cancer is higher than everybody	4	1–5
	17: My relationship with my spouse will be disrupted if I have colon cancer	4	1–5
Barrier	18: I get uncomfortable to talk about colon cancer	4	1–5
	19: I wouldn’t worry about colon cancer if I had regular controls for early diagnosis of colon cancer	2	1–5
	20: Having regular controls for early diagnosis of colon cancer is embarrassing for me	4	1–5
	21: Having regular controls for early diagnosis of colon cancer makes me worry about colon cancer	4	1–5
	22: Having regular controls for early diagnosis of colon cancer takes a lot of time	4	1–5
	23: It is not pleasant to have regular controls for early diagnosis of colon cancer	4	1–5
Health motivation	24: I have a balanced diet	3	1–5
	25: I do exercise at least three times per week	3	1–5
	26: Having regular controls for early diagnosis of colon cancer will help me for early diagnosis of formations that can turn into cancer in the future (polyps, chronic constipation, etc.)	2	1–5
	27: I have regular controls even if I am not sick	3	1–5
	28: It is very costly to have regular controls for early diagnosis of colon cancer	4	1–5
Seriousness	29: The thought of having colon cancer scares me	2	1–5
	30: I would feel better if I had regular controls (check-up) for early diagnosis of colon cancer	2	1–5
	31: My heart beats faster when I think I may have colon cancer	3	1–5
	32: My whole life will change if I have colon cancer	2	1–5
	33: I can’t live more than 5 years if I have colon cancer	4	1–5

The distribution of the points in the HBM subscales for the prevention of colon cancer are given in Table 3.

**Table 3:** Distribution of points in HBM subscales for prevention of colon cancer.

Subscale	Mean	sd	Median	Min	Max
Confidence/benefits	17.1	5.3	16	11.0	37.0
Susceptibility	24.4	3.9	24.0	6.0	30.0
Barriers	20.5	4.1	21.0	8.0	30.0
Health motivation	14.1	2.7	14.0	6.0	25.0
Seriousness	13.7	3.4	14.0	5.0	25.0

Investigating the effect of confounding factors on points, although not statistically significant, confidence/benefit and seriousness scores increased, motivation scores decreased in older age group (*p* = 0.127, *p* = 0.185, *p* = 0.523 respectively). There was a statistically significant difference in barrier scores between age groups (*p* < 0.001). The barrier scores of participants aged 18–29 were significantly higher than those aged 30–39, 40–49, and over 50 (*p* = 0.004, *p* = 0.005, *p* = 0.006 respectively).

When evaluated by gender, both motivation and barrier scores were higher in women than men (*p* > 0.05). There was a difference between the seriousness scores of the female and male participants. The seriousness scores of men were found to be higher than women (*p* < 0.05).

When we looked at the marital status, there was a statistically difference between the barrier scores of the married and single participants. The barrier scores of single participants were significantly higher than married participants (*p* < 0.01). The motivation scores of singles although not statistically significant were also higher than married ones. Also, married people have a slightly higher confidence score (*p* > 0.05).

When we looked at the education, there was a difference between education levels and motivation scores (*p* < 0.05). The motivation scores of high school graduates were higher than university graduates. Susceptibility, seriousness and motivation scores were low in the lower education group. There was a difference between education levels in relation to barriers scores (*p* < 0.05). The barrier scores of the participants whose education level was secondary school and below were found to be significantly lower than the participants with both high school and university degrees (*p* = 0.015, *p* = 0.043 respectively).

When evaluated by work status, although the highest score in confidence and susceptibility belonged to retirees, it was this group with the lowest motivation score. Although there was a statistically significant difference between work status and confidence scores (*p* < 0.05), no difference was found in multiple comparisons.

Although motivation scores were high in housewives and the unemployed, barrier scores were also high. The barrier scores of the students were found to be significantly higher than those of working and retired participants (*p* = 0.001, *p* = 0.013).

In our study we found that age, gender, marital status, educational status and occupational status had no effect on confidence and susceptibility scores. Age, marital status, education and occupation status were found to have an effect on barrier scores. Education status had an effect on motivation scores. Gender was found to affect seriousness scores.

The distribution of the subscales’ points (mean ± sd) are given in Table 4 according to the sociodemographic characteristics.

**Table 4:** Distribution of subscales’ points (mean ± sd) according to the sociodemographic characteristics.

	Confidence	*p* Value	Susceptibility	*p* Value	Barrier	*p* Value	Motivation	*p* Value	Seriousness	*p* Value
Age										
18–29	16.31 ± 5.18	0.127	23.93 ± 3.97	0.115	22.03 ± 3.74	<0.001	14.36 ± 2.65	0.523	13.82 ± 3.44	0.185
30–39	16.82 ± 4.19		25.46 ± 3.54		19.63 ± 4.28	**	14.28 ± 2.64		12.84 ± 3.66	
40–49	18.40 ± 5.42		24.53 ± 3.92		19.60 ± 4.38	**	13.93 ± 3.05		14.29 ± 3.17	
50+	18.09 ± 6.31		23.67 ± 4.57		19.39 ± 3.05	**	13.42 ± 2.78		14.03 ± 3.28	
Sex										
Man	18.02 ± 6.14	0.213	24.11 ± 3.99	0.541	19.89 ± 4.45	0.058	13.92 ± 3.05	0.374	14.50 ± 3.86	0.016*
Woman	16.06 ± 4.60		24.52 ± 3.99		20.98 ± 3.80		14.22 ± 2.57		13.27 ± 3.07	
Marital status										
Single	16.48 ± 4.87	0.125	24.07 ± 4.04	0.329	21.54 ± 3.86	0.001	14.36 ± 2.90	0.306	13.93 ± 3.53	0.428
Married	17.68 ± 5.52		24.61 ± 3.96		19.76 ± 4.09		13.91 ± 2.62		13.56 ± 3.35	
Education										
Secondary school/lower	18.04 ± 4.95	0.533	23.67 ± 4.37	0.352	18.45 ± 3.73	0.020	13.25 ± 3.02	0.021*	12.92 ± 3.36	0.403
High school	17.19 ± 5.56		24.07 ± 4.47		21.07 ± 4.44	*	14.69 ± 2.54		13.99 ± 3.54	
University	16.89 ± 5.10		24.75 ± 3.48		20.66 ± 3.73	*	13.86 ± 2.79		13.70 ± 3.36	
Work status										
Working	17.59 ± 5.59	0.029 NS	24.77 ± 3.89	0.144	19.97 ± 4.34	0.001	14.07 ± 2.93	0.128	13.83 ± 3.59	0.917
Student	15.46 ± 4.59		23.27 ± 4.46		22.43 ± 3.57	**	14.27 ± 2.39		13.73 ± 3.34	
Retired	18.53 ± 5.07		24.95 ± 4.03		19.21 ± 2.57	*	13.05 ± 2.70		13.53 ± 3.61	
Housewife + Unemployed	17.84 ± 4.21		24.47 ± 2.46		20.26 ± 3.38		15.00 ± 2.47		13.26 ± 2.51	

**Notes:**

* *p* < 0.05.

** *p* < 0.01.

*p* values for age, sex, marital status, education, work status respectively; 0.04, 0.25, 0.06, 0.30, 0.12 for confidence; 0.11, 0.47, 0.32, 0.33, 0.15 for susceptibility; <0.001, 0.05, 0.001, 0.02, 0.001 for barrier; 0.39, 0.48, 0.26, 0.03, 0.28 for motivation; 0.18, 0.01, 0.43, 0.40, 0.94 for seriousness.

## Discussion

HBM is based on the idea that there is a correlation between the beliefs and behaviors of individuals and it is frequently used to explain preventive health behavior. It is viewed as an effective guide that explains and measures behaviors to protect and improve health, as well as what motivates or prevents patients in relation to compliance with therapy in various health problems (Gözüm & Çapık, 2014; Yılmaz, Dereli & Yelten, 2016; Champion, 1984; Champion & Scott, 1997; Jacobs, 2002; Aydoğdu & Bahar, 2011; Ozsoy, Ardahan & Ozmen, 2007; Janz, Champion & Strecher, 2002).

This study was conducted in a quaternary reference hospital in the capital city of Turkey, for the purpose of evaluating the health beliefs about CRC. In line with our prediction made before the study, the participants’ average age was 35 and most of them were female. In addition, those over 50 years applied to our outpatient clinic who had no idea about CRC and never had colonoscopy before, were also included after being informed about the study. Thus, it was aimed to raise awareness among this group as well. They constituted 15% of the study participants.

In previous studies, the selected age groups were mostly over 50, which is the age that screenings for early diagnosis begin (Baysal & Turkoglu, 2013; Sohler, Jerant & Franks, 2015). Our study is different in that it included participants as young as 18 years old, allowing for intervention at an early age.

Confidence-benefit perception refers to the belief/expectation of the individual that the risk of the disease occurring will decrease as a result of a certain behavior, which is the “perceived benefit” (Aydoğdu & Bahar, 2011). It is the perception of benefit that determines whether the person will be open to implementing the health behavior throughout her/his life.

The average number of points our participants received in the confidence-benefit perception subscale was 17.1 ± 5.3. This is closer to the lower end of min-max points (Gözüm & Çapık, 2014; Yılmaz, Dereli & Yelten, 2016; Champion, 1984; Champion & Scott, 1997; Jacobs, 2002) which shows that the benefit perception for the prevention of CRC is low. The 40–49 age group had the highest points in the benefit subscale. In a similar study by Baysal & Turkoglu (2013) with participants 50 years and older, the average score of the benefit subscale of HBM was 42.38 ± 9.02 points which is much higher than in our study. In another study by Nar based on 400 individuals whose first degree relatives had had a CRC diagnosis, the confidence-benefit average points were 48.9 ± 5.1, which is higher than both our and Turkoglu’s studies. This may be due to the fact that their study population included more people who had had first degree relatives diagnosed with CRC than ours. In both studies carried out in Turkey, confidence-benefit, health motivation and seriousness perception average scores were higher, whereas susceptibility and barrier points were lower than ours. This difference may be due to the younger age of our participants and the fact that other study groups had been composed of first degree relatives of patients with CRC. Susceptibility perception includes acceptance of the diagnosis by the patient and the possibility of the disease happening. The possibility of an individual to show risk reduction behavior increases as the perception of susceptibility increases (Aydoğdu & Bahar, 2011). Our participants’ average points (24.4) are closer to the maximum (min–max: 6–30). This is higher than the average points in both Baysal & Turkoglu (2013) and Nar (2010) studies. This difference again may be due to the younger age of our participants. Barrier perception is related to the factors that prevent or complicate the exhibition of a preventive health behavior. Individuals evaluate the positive or negative results of such behavior for themselves. According to study results, barrier perception is the most critical factor for the exhibition of the behavior (Glanz, Rimer & Viswanath, 2008) and the difference between barrier and benefit perceptions is seen as the most important variable complicating the exhibition of preventive health behaviors. Also, the perception of susceptibility, seriousness and benefits should have a reducing effect on the perception of barriers for the realization of the behavior (Jacobs, 2002). In our study, the “18–29 age group” and the “singles group” had the highest points in the barrier subscale. Moreover, as the motivation score was found to be high in young and women, this may help changing the misbeliefs and reducing the barrier scores.

The average of our study group for the barrier subscale was 20.5 ± 4.1(min–max: 6–30). This value was found to be 15.6 ± 4.3 in Baysal’s and 15.2 ± 3.8 in Nar’s studies.

Health motivation covers the individual’s self belief, determination and will power for the exhibition of the behaviors needed to reach the expected outcome, so it plays an important role in initiating the behavioral change and maintaining it (Glanz, Rimer & Viswanath, 2008). The most important factor that affects the individual’s perception of self efficacy is the real performance. The perception of self efficacy increases if the individual can repeatedly exhibit certain behaviors, and decreases if the exhibition fails repeatedly. In the motivation subscale, the average of our participants’ points was 14.1± 2.7 (min and max points are 5–25). It was more than 15 in both Baysal’s and Nar’s study.

The notion that expresses personal beliefs about the severity of the disease is “seriousness perception”. Although this perception mostly depends on medical knowledge or experience, it may also arise from beliefs about the difficulties that the disease will create for the person or the general effects on the person’s life. Sung et al. (2008) showed that seriousness and barrier perceptions have a significant effect on an individual participating in screening tests for cancer. In this subscale men have significantly higher mean points than women (14.51 ± 3.87). Min–max points are 5–25 and our mean was 13.7 ± 3.4. This is below both Baysal’s (16.5 ± 4.0) and Nar’s studies (16.5 ± 4.3).

There is a statistically significant difference in terms of barrier scores of the subscale and age groups, especially with a negative effect on the young age group.

This data supports our hypothesis and reveals the necessity to change the perception of the young age group about barriers. Being married or single also made a significant difference in relation to barrier scores. The barrier scores were already high in the young group, and when we consider that the majority of this group consists of singles, it is expected that the barrier would be high among single participants. As married group had lower barrier scores and higher confidence scores, this combination may facilitate increasing the motivation.

There was a significant difference between education levels in terms of barriers scores. Interestingly, the higher the education level, the higher the barrier score. Increased education level does not affect taking early measures and overcoming barriers for CRC prevention. Besides, the motivation scores of high school graduates were higher than university graduates.

When we look at the work status, student participants’ barrier scores were higher than those who had a job and had retired. The barrier scores of student group were found to be significantly higher than those of working and retired participants (*p* = 0.001, *p* = 0.013). Because the age has a strong relation to the work status, students are usually much younger. In the “18–29 age group” mostly students had the highest points in the barrier subscale. In other words, both being in the young age group and being a student pose a high risk in terms of barrier.

The seriousness scores of men were found to be higher than women. This result is not compatible with our hypothesis.

According to our results, even being over 40 years old does not statistically increase confidence perception in a significant way. Being below 30 years old, being female, being single, and being high school graduate or higher does increase barrier perception, while being male increases seriousness perception.

Although a well organized screening program is mediated through the Ministry of Health for CRC in primary health care facilities in Turkey, the habit of complying with this is insufficient. Many people resist screening decisions.

This study shows that the benefit perception for the prevention of CRC is lowest among young people. This means that the person is not open to implementing the health behavior in her/his life. Meanwhile, the barrier subscale has high points among the young ones, single ones, high school graduates and students.

## Conclusion

Our study points out the fact that attitudes about preventive health measures are mainly affected by age, gender, education level and marital status. These personal characteristics should be taken into consideration while offering screening programs and preventive health measures to individuals for protection from colorectal cancer.

As a conclusion we can say that it may be better to give specific messages and specific barriers according to personal characteristics instead of general messages about conducting screening tests beginning from youth.

## Supplemental Information

10.7717/peerj.10951/supp-1 Supplemental Information 1 Raw data.

10.7717/peerj.10951/supp-2 Supplemental Information 2 Variables (in English).

## Additional Information and Declarations

### Data Availability

The following information was supplied regarding data availability: Raw data are available in the Supplemental Files.
